# Efficacy and Safety in Dogs Following Administration of an Alphavirus RNA Particle Canine Influenza H3N2 Vaccine

**DOI:** 10.3390/vaccines12101138

**Published:** 2024-10-04

**Authors:** Haley M. Classe, Jennifer C. Dant, Mark Mogler, Kenneth A. Stachura, Rhonda L. LaFleur, Zach Xu, Ian Tarpey

**Affiliations:** 1 Research and Development Department, Merck Animal Health, Elkhorn, NE 68022, USA; 2 Research and Development Department, Merck Animal Health, Ames, IA 50010, USA; 3 Research and Development Department, MSD Animal Health, 5831 AN Boxmeer, The Netherlands

**Keywords:** canine, influenza, H3N2, vaccine, efficacy, safety, shedding

## Abstract

Canine influenza virus (CIV) H3N2 causes a highly contagious respiratory disease in dogs and has been the source of outbreaks across North America since 2015. An injectable RNA Particle (RP)-CIV H3N2 vaccine has been developed to protect dogs against this disease. To demonstrate efficacy, dogs were randomized into two treatment groups, then vaccinated subcutaneously twice, 21 days apart, with a placebo vaccine (*n* = 20) or an RP-CIV H3N2 vaccine (*n* = 20). Three weeks later, dogs were challenged intranasally with virulent CIV H3N2 and observed daily for 10 days for clinical signs of disease. Nasal swabs were also collected daily to evaluate the shedding of the challenge virus. Ten days post-challenge, the dogs were euthanized, and the lungs were examined for consolidation. RP-CIV H3N2 vaccination demonstrated a significant reduction in the duration of clinical signs, duration and amount of virus shed, lung consolidation, and the incidence of suppurative pneumonia. To evaluate safety, dogs from multiple geographic regions were vaccinated subcutaneously, 3–4 weeks apart, with an RP-CIV H3N2 vaccine and observed for adverse events for 14 days after each administration. The RP-CIV H3N2 vaccine was deemed safe, with lethargy being the most reported adverse event at a rate of 1.6%.

## 1. Introduction

Canine influenza is an easily transmissible respiratory disease that is caused by influenza A viruses, primarily avian-origin canine influenza virus (CIV) H3N2 [1]. CIV is transmitted via direct contact or airborne particles for up to ten days post-infection, while the stability of the virus outside of a host is just one to two days [2,3]. Although dogs are the primary hosts for CIV, interspecies transmission puts cats, ferrets, horses, mice, guinea pigs, and other species at risk for infection as well [4,5,6]. Animals in close contact are, therefore, at increased risk for exposure, such as those in shelters, boarding kennels, or dog parks [6]. Since 2015, CIV H3N2 outbreaks have been an issue throughout North America and Asia [4,7,8]. Dogs with CIV H3N2 infection can exhibit clinical signs, including cough, nasal discharge, depression, and occasionally fever [6]. In more severe cases of infection, suppurative bronchopneumonia can develop, which can be fatal [9]. Additionally, since CIV H3N2 is associated with canine infectious respiratory disease complex (CIRDC), the probability of coinfections in these cases is even greater. For these reasons, it is highly recommended by the American Animal Hospital Association to vaccinate dogs for CIV to prevent the spread of disease and possible epidemics.

Alphavirus-derived RNA particles (RP), particularly those based on Venezuelan equine encephalitis virus (VEEV), are a compelling platform for the development of vaccines [10,11]. The RP utilizes alphavirus coat proteins to deliver a propagation-defective, self-amplifying RNA to cells in vivo, where the viral replicase drives RNA amplification and efficient protein expression of inserted antigen-coding sequences [12]. The use of the attenuated VEEV strain TC-83-derived sequences and promoterless split helper production systems reduce the biocontainment requirements for manufacturing RP vaccines [12,13]. The TC-83-derived RP vaccine platform has demonstrated efficacy against influenza virus challenge in multiple naturally susceptible species, including swine, chickens, ducks, and turkeys [14,15,16,17,18,19]. Accordingly, we used this TC-83-derived RP vaccine platform as an innovative approach to develop a new CIV H3N2 vaccine. Traditional inactivated CIV vaccines have required the use of adjuvants to assist in accentuating the immune response, but this heightened inflammatory reaction can sometimes be deleterious and may not drive a balanced immune response. In contrast, the RP platform allows for this new CIV H3N2 vaccine to be adjuvant-free while eliciting a robust humoral and cell-mediated immune response by targeting dendritic cells. In addition, the small amount of RP that is required for an efficacious dose allows for a 0.5 mL administration volume, which can provide a more comfortable vaccination experience, especially for smaller breeds. Furthermore, the RP platform allows for a more closed system manufacturing process, leaving the final vaccine formulation preservative and thimerosal-free. Together, we describe the studies demonstrating the safety and efficacy of this innovative CIV H3N2 vaccine.

## 2. Materials and Methods

### 2.1. Ethics

All dog studies that used purpose-bred animals were approved prior to study initiation and were in compliance with Institutional Animal Care and Use Committee (IACUC) guidelines (protocols E-2023-28 and CVR-014-01).

### 2.2. Vaccine Formulations

The propagation-defective replicon RNA particles contain alphavirus replicon RNA expressing a Type A Canine Influenza H3N2 hemagglutinin (HA) gene packaged with the capsid protein and glycoproteins of the avirulent TC-83 strain of VEEV. The wild-type Canine Influenza H3N2 HA sequence was obtained from a 2015 Iowa strain (A/canine/Iowa/22619-4/2015(H3N2)), and a synthetic, codon-optimized gene for the HA open reading frame and flanking restriction sites was prepared by a supplier (ATUM, Newark, CA, USA). The synthetic gene was cloned into the DNA plasmid vector, pVHV, which is derived from the avirulent human vaccine strain of VEEV (strain TC-83). Batches of experimental RPs were prepared as previously described [20]. Briefly, Vero cells were electroporated with individual replicon RNA and promoterless helper RNAs encoding the VEEV strain TC-83 glycoproteins and capsid. The RPs were combined with stabilizer [NZ Amine, sucrose, and Dulbecco’s Modified Eagles Medium (DMEM)], lyophilized, and reconstituted with 0.5 mL vaccine diluent prior to use. The placebo vaccine used in the efficacy study consisted of all components of the test vaccine except the RP-CIV H3N2 antigen. 

### 2.3. Challenge Material

The heterologous canine influenza H3N2 challenge strain was originally isolated in eggs from a field sample collected in 2017 from a dog in Louisville, Kentucky, suffering from canine respiratory disease. On the day of the challenge, the frozen challenge virus was thawed and diluted in sterile, cold DMEM. The challenge dose (4.5–8.2 log_10_TCID_50_/dose) was optimized in preliminary studies to generate clinical signs of disease and lung consolidation in at least 50% of the animals.

### 2.4. Efficacy Study Design

Seven- to eight-week-old conventional, purpose-bred beagles that had not been exposed to or vaccinated for CIV were enrolled in this study. These dogs were housed in an isolation facility in barrier rooms and randomized into two treatment groups using litter size and housing units as randomization factors. All dogs were vaccinated subcutaneously twice, 21 days apart (study days 0 and 21), with a 0.5 mL dose of RP-CIV H3N2 vaccine (*n* = 20) at a minimum protective dose or a 0.5 mL dose of a placebo vaccine that consisted of all components of the test vaccine except the RP-CIV H3N2 antigen (*n* = 20). Rectal temperatures were recorded on the day prior to each vaccination, on the day of each vaccination prior to administration, and for 2 days following each vaccination. In addition, all dogs were monitored for injection site reactions starting at 2–4 h post-vaccination and for 2 days post-vaccination. Injection sites were assessed for visibility, size, and description, including thickening, soft (edema), hard, tender, visible, or scratching at the injection site. Whole blood for serum was collected on the day prior to each vaccination (study days −1 and 20) and then on study days 28, 33, and 40 (the day before challenge). Nasal swabs were also collected on study day 40. 

Three weeks following the second vaccination, 19 vaccinated dogs and 20 placebo-vaccinated control dogs were challenged by the intranasal route with virulent CIV H3N2. One vaccinated dog had to be humanely euthanized prior to challenge due to welfare concerns following a blood collection-associated adverse event. During the 10-day post-challenge observation phase of this study, nasal swabs were collected daily, and clinical signs, including spontaneous coughing, retching, sneezing, nasal discharge, dyspnea, and fever (rectal temperature ≥ 103.5 °F), were monitored daily. Dogs were humanely euthanized on day 10 post-challenge, and the lungs were immediately evaluated for consolidation. Each lobe of the right (cranial, middle, caudal, and accessory lobes) and the left (cranial-cranial, cranial-caudal, and caudal lobes) lungs were scored individually for percent consolidation by a veterinarian blinded to the treatment group designation of each dog. The percent consolidation of each lung lobe was then converted into a weighted score, and a total score for each dog was calculated [21]. The weighted lung lesion score was based on the size of each lobe: right cranial (×0.152), right middle (×0.1), right caudal (×0.248), accessory lobes (×0.09), left cranial-cranial (×0.091), left cranial-caudal (×0.06), and left caudal (×0.259). Lung swabs and lung tissue were also collected from each dog.

### 2.5. Immunofluorescence Assay for Vaccine Titer

The titer of the RP-CIV H3N2 vaccine was determined by serially diluting the vaccine, which was added to a Vero cell monolayer culture in 48-well plates (Greiner; Monroe, NC, USA) and incubated at 37 ± 1 °C in 4–6% CO_2_ for 18–22 h. After incubation, the cells were fixed and stained with the primary antibody specific for CIV H3N2 HA followed by an Alexa Fluor^®^ 488 conjugated secondary antibody (Invitrogen; Carlsbad, CA, USA). RPs were quantified by counting positive, fluorescent-stained cells. The titer was calculated from the known dilution and inoculation volume.

### 2.6. Hemagglutination Inhibition Assay for Serology

Whole blood collected into serum separation tubes (BD; Franklin Lakes, NJ, USA) was allowed to clot for a minimum of 2 h at 15–30 °C, then was centrifuged at 1000× *g* to separate the serum. Serum samples were tested in a hemagglutination inhibition assay (HAI) to evaluate the serological response post-vaccination. Briefly, CIV antigen was added to 1:2 dilutions of the test serum. Following incubation, a 0.5% rooster red blood cell suspension was added to the serum/virus mixtures. After another incubation, the results were read, and the titer was recorded as the reciprocal of the highest dilution of serum showing hemagglutination inhibition.

### 2.7. Canine Influenza Virus Titration for Viral Shedding

Polyester fiber tipped swabs (Fisherbrand; Pittsburgh, PA, USA) were wetted in transport media [DMEM (Corning; Corning, NY, USA), gentamicin (Gibco; Grand Island, NY, USA), and amphotericin-B (Corning; Corning, NY, USA)] and inserted into each nostril of the dog. In the lab, swabs were removed from the transport media, and the liquid contents were centrifuged at 500× *g*. The supernatant was used to inoculate a confluent monolayer of canine kidney cells that were planted on 96-well tissue culture plates (Falcon; Corning, NY, USA). Plates were incubated at 36 ± 2 °C in 4–6% CO_2_ for 7 days, and monolayers were then observed for cytopathic effect. The virus titer was calculated by the Spearman–Karber method.

### 2.8. Lung Swab Testing

Polyester fiber-tipped swabs (Fisherbrand; Pittsburgh, PA, USA) were used to swab the interior of the lung tissue and placed into collection media. One swab was placed into supplemented media containing tryptose phosphate broth (BD; Franklin Lakes, NJ, USA) and glycerol (Fisher; Hampton, NH, USA), which was tested for *Bordetella bronchiseptica* (*B. bronchiseptica*) and *Streptococcus equi* subspecies *zooepidemicus* (*S. equi* subsp. *zooepidemicus*). For *B. bronchiseptica*, the lung swab material was streaked onto MacConkey agar plates (Thermo Scientific; Waltham, MA, USA), and the plates were incubated at 36 ± 2 °C for 48–96 h. For *S. equi* subsp. *zooepidemicus*, the lung swab material was streaked onto anaerobic reducible blood agar with colistin and nalidixic acid agar plates (Thermo Scientific; Waltham, MA, USA), and the plates were incubated at 36 ± 2 °C for 24–48 h. The second swab was placed into supplemented media containing trypticase soy broth (BD; Franklin Lakes, NJ, USA), glycerol (Fisher; Hampton, NH, USA), and carbenicillin (Teknova; Hollister, CA, USA), which was tested for *Mycoplasma* by streaking the lung swab material onto PPLO agar plates (Thermo Scientific; Waltham, MA, USA) and incubating at 36 ± 2 °C in 4–6% CO_2_ for 21 days. The third swab was placed into supplemented media containing Dulbecco’s Modified Eagles Medium (Corning; Corning, NY, USA), gentamicin (Gibco; Grand Island, NY, USA), and amphotericin-B (Corning; Corning, NY, USA), which was tested for canine parainfluenza (CPI) virus. Dilutions of lung swab material were inoculated onto dog kidney cells, and after 4–6 days, monolayers were fixed and stained with fluorescein-conjugated CPI antiserum (VMRD; Pullman, WA, USA). Titers were calculated by the Spearman–Karber method. 

### 2.9. Histopathology of Lung Tissue

Fresh lung tissue was collected after lung scoring and placed into 10% buffered formalin (Fisherbrand; Pittsburgh, PA, USA) for histopathology testing performed by the University of Nebraska-Lincoln Veterinary Diagnostic Center. The slides were read by a board-certified pathologist (American College of Veterinary Pathologists) who was blinded to the treatment group information. The lesion types were assigned a score, and the nature of the lesion was described as suppurative or not. 

### 2.10. Statistical Analysis

Statistical analysis was performed in R-3.5.3 and Microsoft^®^ Excel^®^ version Microsoft 365^®^ to evaluate efficacy. The duration of cough, duration of viral shedding, and lung scores were compared between treatment groups by the Wilcoxon Rank Sum Test. The proportion of affected dogs with pneumonia in the two groups was calculated and compared using Fisher’s Exact Test. Statistical significance for serologic geometric means, clinical sign scores, and viral load were calculated and compared using a two-tailed Welch’s T-test. Significance was declared for two-sided *p*-values < 0.05. 

### 2.11. Field Safety Study Design

A total of 654 dogs (330 males; 324 females) were enrolled from 5 different geographic locations, and 644 dogs completed this study. The ages of the dogs ranged from 8 weeks to 15 years, with 210 being purpose-bred beagles and 434 being client-owned dogs of various breeds. A total of 217 dogs (217; 33%) were 8 weeks of age at the time of vaccination. Owners were informed of the details of this study and signed a consent form prior to enrollment. 

Each dog was vaccinated by the subcutaneous route with a 0.5 mL typical field dose vaccine on study day 0 and then another 0.5 mL dose was administered 3–4 weeks later for a total of 1301 doses administered. Owners were instructed to closely observe their dog(s) daily for 14 days following the first and second vaccinations and document any adverse local or systemic events in a diary. An adverse event was defined as any observation in the dogs, whether considered to be product-related or not, that was unfavorable and unintended and occurred after the use of the test vaccine. A serious adverse event was defined as an adverse event that was fatal or life-threatening; resulted in significant disability, incapacity, or a congenital anomaly/birth defect; or resulted in permanent or prolonged signs or required professional intervention beyond routine prevention measures and common first aid. The relationship to the vaccine was evaluated as “related” or “not related” by a study veterinarian. 

## 3. Results

### 3.1. Serology

**Figure 1:**
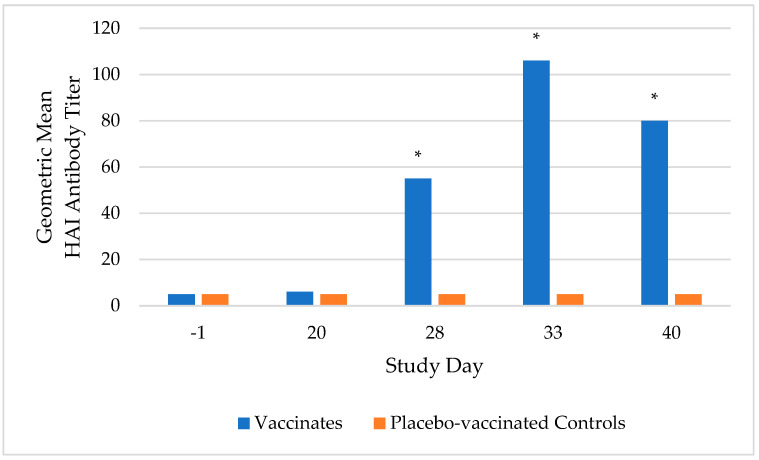
Whole blood for serum was collected on study days −1, 20, 28, 33, and 40 to monitor serological titers to canine influenza virus (CIV) H3N2. All vaccinated dogs (*n* = 20) sero-converted following the second vaccination, with the highest hemagglutination inhibition (HAI) antibody titers occurring on study day 33. The test vaccine significantly increased serological titers on study days 28, 33, and 40 (* *p* < 0.0001) compared to the placebo-vaccinated controls (*n* = 20).

Prior to vaccination, all dogs were sero-negative (HAI antibody titer < 10) to CIV H3N2, indicating they were susceptible to vaccination. Following the second vaccination, all vaccinated dogs sero-converted, with the highest HAI antibody titers occurring on study day 33, ranging from 10 to 320 with a geometric mean of 106. Meanwhile, the placebo-vaccinated control dogs remained sero-negative until challenge. On study days 28, 33, and 40, the geometric mean titer was significantly higher (*p*-value < 0.0001) in the vaccinated group compared to the placebo-vaccinated control group (Figure 1). 

### 3.2. Post-Challenge Monitoring

**Figure 2:**
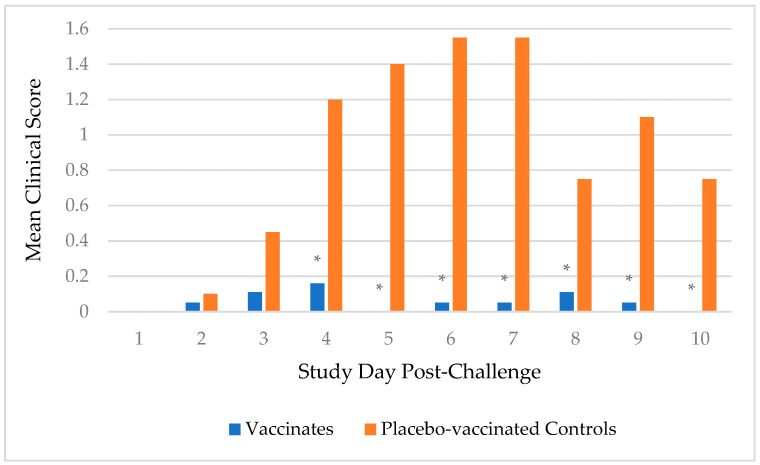
Dogs were observed for clinical signs of CIV H3N2 and given a score. The test vaccine (*n* = 19) significantly reduced the clinical signs of disease on days 4–10 following virulent challenge (* *p* ≤ 0.006) compared to the placebo-vaccinated controls (*n* = 20).

Following challenge, fevers were sparse with only one placebo-vaccinated control dog having a fever (104.2 °F and 103.6 °F) on 2 separate days, and one vaccinated dog having a fever (103.5 °F) on one day. Spontaneous cough, with or without retching, was the primary clinical sign for analysis, but other clinical signs of respiratory disease such as sneezing and serous nasal discharge were also observed. Of the 20 placebo-vaccinated control dogs, 18 (90%) coughed on multiple days and 1 (5%) coughed on 1 day. In contrast, only 1 (5%) of the 19 vaccinates coughed on multiple days and another 5 (26%) coughed on 1 day. In addition, the overall duration and severity of clinical signs were more apparent in the placebo-vaccinated control dogs compared to the vaccinated dogs. Duration of coughing (in days), from first to last occurrence, was determined for each dog. The median duration of coughing for the placebo-vaccinated control group was 6 days, which was significantly more than the 0 days for the vaccinated group (*p*-value < 0.0001). Furthermore, a scoring system was implemented to evaluate clinical signs. Sneezing, serous nasal discharge, and spontaneous coughing were all assigned a score of 1, while spontaneous cough with retching was assigned a score of 2. For dogs with multiple clinical signs on a given day, the scores were added together. The mean clinical sign scores were significantly higher, and thus more severe, in the placebo-vaccinated control group on study days 4 (*p*-value = 0.001), 5, 6, 7 (*p*-value < 0.0001), 8 (*p*-value = 0.006), 9 (*p*-value = 0.0005), and 10 (*p*-value = 0.0008) post-challenge compared to the vaccinated group (Figure 2).

### 3.3. Viral Shedding

**Figure 3:**
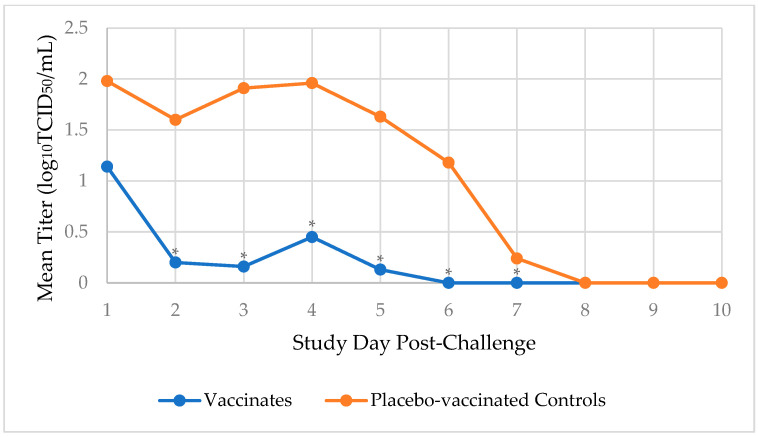
Nasal swabs were collected daily post-challenge and titrated for CIV H3N2 virus. The daily mean titer for the vaccinated dogs (*n* = 19) and the placebo-vaccinated controls (*n* = 20) was calculated and compared. The test vaccine significantly reduced the amount of CIV H3N2 virus that was shed from dogs on days 2–7 following virulent challenge (* *p* ≤ 0.02).

Prior to challenge, dogs were not shedding any CIV, indicating they were susceptible to the challenge virus. After the CIV H3N2 challenge, the duration of viral shedding (in days), from the first to the last occurrence, was determined for each dog. Virus shedding peaked 1 day after challenge, and the median number of days during which the virus was shed in the placebo-vaccinated control group was 6 days, which was significantly more than the 1 day in the vaccinated group (*p*-value < 0.0001). By day 6 post-challenge, none (0%) of the 19 vaccinated dogs were shedding the virus, compared to 18 (90%) of 20 placebo-vaccinated control dogs who were still shedding a mean viral load of 1.18 log_10_TCID_50_/mL. The viral load was significantly higher in the placebo-vaccinated control dogs on days 2, 3, 4, 5, 6 (*p*-value < 0.0001), and 7 (*p*-value = 0.02) post-challenge compared to the vaccinated dogs (Figure 3). 

### 3.4. Lung Consolidation and Histopathology

**Figure 4:**
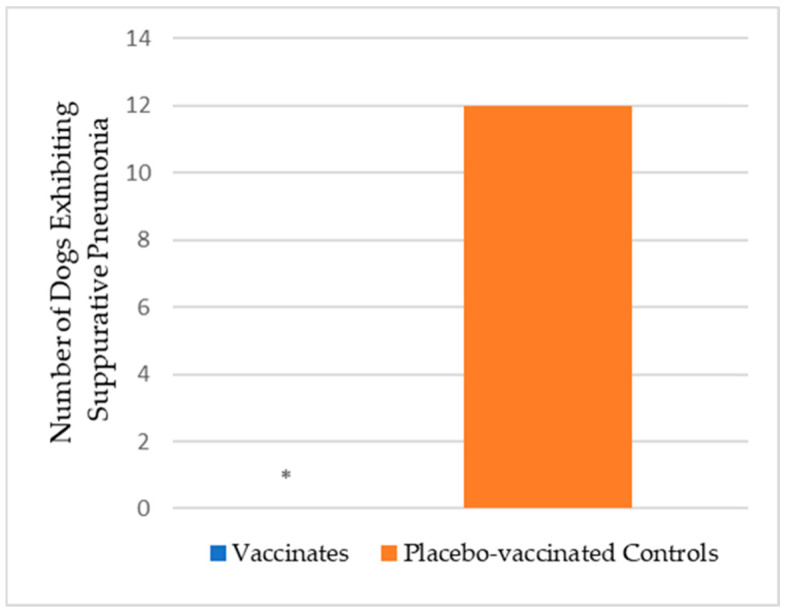
Lung tissue was collected for histopathology. The test vaccine (*n* = 19) significantly reduced the number of dogs exhibiting signs of suppurative pneumonia (* *p* <0.0001) compared to the placebo-vaccinated controls (*n* = 20).

**Figure 5:**
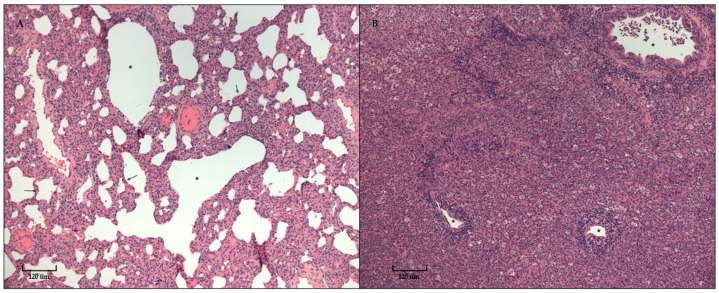
Image **A** (**left**) represents lung tissue from a vaccinated dog; bronchioles (*) are clear and alveolar walls (arrows) are thickened slightly by atelectatic artifact but are normal. In contrast, Image **B** (**right**) represents lung tissue from a placebo-vaccinated control dog with greater than 50% lung consolidation indicative of suppurative pneumonia; bronchioles (*) vary from clear to obstructed with neutrophils and macrophages, the alveoli are filled with neutrophils and macrophages that obscure alveolar walls near margins of complete consolidation, and alveolar walls are thickened by macrophage and neutrophil infiltrates.

All dogs were necropsied 10 days post-challenge to examine the lungs and score for consolidation. The range of weighted lung scores for the placebo-vaccinated control dogs was 0 to 22.6, with a median of 7.2. In contrast, the range of weighted lung scores for the vaccinated dogs was 0 to 1.0, with a median of 0 (*p*-value < 0.0001). Lung tissue was collected at the time of necropsy, preserved in formalin, and sent to the University of Nebraska-Lincoln for histopathology. A total of 12 (60%) of 20 dogs in the placebo-vaccinated control group exhibited varying degrees of suppurative pneumonia, which was significantly more (*p*-value < 0.0001) than none of the dogs in the vaccinated group (Figure 4). The degree of suppurative pneumonia was divided as either less than 50% (2 of 12 affected dogs) or greater than 50% (10 of 12 affected dogs) of the lung displaying severe consolidation (Figure 5). The lung consolidation and suppurative pneumonia were a direct result of CIV H3N2 infection, as the lung swabs collected during necropsy were negative for many of the CIRDC-associated pathogens such as *B. bronchiseptica*, *Mycoplasma*, *S. equi* subsp. *zooepidemicus*, and CPI. 

### 3.5. Vaccine Safety

In this efficacy study, there were no injection site reactions or systemic adverse events following either vaccination. In addition, none of the dogs ran a fever following vaccination. In the 654-dog field safety trial (1301 doses), there were 117 adverse events documented during this study, with 85 [occurring in 64 (9.8%) dogs] of them considered “not related” to the test vaccine and 32 [occurring in 24 (3.7%) dogs] considered to be “related” to the test vaccine. Lethargy was the most common adverse event attributed to the test vaccine at a rate of 1.6%. Other adverse events included diarrhea (0.3% of doses) and polydipsia (0.2% of doses). There were only two adverse events associated with the injection site, including swelling (0.1% of doses) and pain (0.1% of doses), which resolved by the next day. Finally, there was one adverse event characterized as anaphylaxis that presented as lethargy and excessive salivation, which resolved promptly following treatment with diphenhydramine and dexamethasone. 

## 4. Discussion

A novel RNA Particle platform has been used to develop a new CIV H3N2 vaccine for dogs. The vaccine has been shown to be very safe and highly efficacious. This efficacy study was valid, as all dogs were sero-negative prior to vaccination, the placebo-vaccinated control dogs remained sero-negative until challenge, all dogs were negative for CIV H3N2 shedding prior to challenge, and the virulent CIV H3N2 challenge material induced severe respiratory disease in the placebo-vaccinated control group. The complete data package demonstrates strong protection against the challenge in dogs vaccinated with the non-adjuvanted RP-CIV H3N2 vaccine at a minimum protective dose. The minimum protective dose establishes the lowest amount of RNA particles needed in the vaccine to provide protection against the virulent challenge and serves as the baseline to ensure all commercially available vaccines are above this minimum threshold.

The RP-CIV H3N2 vaccine protected dogs by significantly reducing the duration of viral shedding, the amount of virus shed, the duration of coughing, the severity of clinical signs, the incidence of suppurative pneumonia, and the development of lung consolidation. CIV H3N2 shedding and viral load were determined by quantifying the amount of virus in nasal swabs collected from dogs daily for 10 days post-challenge. The median duration of shedding (in days) was significantly lower in vaccinated dogs compared to the placebo-vaccinated control dogs. In addition, viral shedding from the vaccinates had completely subsided 2 days sooner than for the placebo-vaccinated controls. Furthermore, the amount of virus that was shed in the placebo-vaccinated control dogs was significantly higher over 6 days post-challenge than in the vaccinated dogs. Therefore, vaccination with the RP-CIV H3N2 vaccine not only reduces the number of days that the virus is shed, but it also reduces the amount of virus shed. This is important in reducing the risk of transmission from an infected dog to a naïve animal such as other pets or animals in close contact at a shelter or dog park. One of the limitations of this study is the use of purpose-bred animals, which largely removes the influence of other variables on the assessment of vaccine efficacy. Purpose-bred dogs have similar genetic backgrounds, environmental factors, vaccination history, and lack of exposure to other pathogens. This study design allows for a more accurate evaluation of the differences between treatment groups but does not necessarily correlate to pet dogs that are of different ages, breeds, environments, and risk to a variety of pathogens. 

The primary clinical sign of CIV H3N2 infection is coughing. In the current study, 6 (32%) of the 19 vaccinated dogs coughed on just one or two occasions, while 19 (95%) of the 20 placebo-vaccinated control dogs persistently coughed anywhere from 1 to 8 days. The median duration of coughing was significantly lower in the vaccinated group (*p*-value < 0.0001). Additionally, the severity of clinical signs in the placebo-vaccinated control group was significantly more than that of the vaccinated group. For example, only 1 (5%) of the vaccinated dogs retched once, while 18 (90%) placebo-vaccinated control dogs retched persistently. Furthermore, nasal discharge was only present in the placebo-vaccinated control group. Taken together, clinical signs of a CIV infection are significantly reduced in both duration and severity due to vaccination with the RP-CIV H3N2 vaccine even after a highly virulent CIV H3N2 challenge. Consequently, morbidity can be diminished or possibly even prevented using this vaccine.

Because CIV H3N2 causes a lower respiratory infection in dogs, lung consolidation is an important disease outcome and is the primary variable that the USDA uses when evaluating CIV studies. Consolidation occurs when exudate from the infection fills the air spaces within the lung lobes, resulting in the clinical signs of coughing and retching. In addition, these infections can often result in pneumonia, which can be fatal. Pneumonia can put the welfare of an infected dog at great risk by increasing the severity of clinical signs as well as increasing the risk of coinfections from other viral and bacterial CIRDC pathogens. In the current study, the median weighted lung score of the vaccinated group was significantly lower than the placebo-vaccinated control (*p*-value < 0.0001). Fourteen (74%) dogs that had been vaccinated with the RP-CIV H3N2 vaccine did not have any consolidation on any lung lobe, and the highest total lung score for the group was 1.0. In contrast, only 2 (10%) of the placebo-vaccinated control dogs had a lung score of 0, and the highest score for the group was 22.6. The higher lung scores in the placebo-vaccinated control group correlated to 60% of the dogs affected with suppurative pneumonia. Additional testing confirmed that the lung consolidation and clinical disease observed in this study were due to CIV H3N2 and no other CIRDC-associated pathogens. In summary, vaccination with the RP-CIV H3N2 vaccine at minimum protective dose is efficacious in dogs as young as 8 weeks of age, as demonstrated by the significant reduction in lung consolidation and the incidence of suppurative pneumonia. Another limitation of this study is that vaccinated dogs are evaluated using a very specific challenge model that was designed and validated so that the lungs were scored during peak consolidation (as per regulatory guidance). Therefore, the efficacy profile of the vaccine is not fully understood, but overall, the vaccine demonstrated solid protection under a worst-case situation (minimum protective dose of antigen in the vaccine and high amount of virulent CIV administered up the nose at challenge). 

Demonstrating vaccine safety in the target animal is another aspect of vaccine development. In the current efficacy study, there were no local or systemic adverse events recorded. No swelling or tenderness at the injection site was observed upon palpation of each dog on the days of and after each vaccination. In addition, a field safety study was conducted in purpose-bred dogs, as well as client-owned dogs, to further evaluate the safety profile of this vaccine. Of the 1301 administrations of 2 different serials of the RP-CIV H3N2 vaccine containing an amount of antigen representative of the vaccine to be used by customers, only 2 caused a local reaction that resolved within 24 h without treatment. Overall, only 2.5% of the doses administered in the field safety trial caused adverse reactions that were primarily mild and of short duration. Lethargy was the most common event (1.6% of total administrations). In addition, the safety profile of the RP-CIV H3N2 vaccine appears to be no different in younger dogs than older dogs, as well as males versus females. The combined data demonstrates that the RP-CIV H3N2 vaccine is safe for use in dogs as young as 8 weeks of age.

In summary, a new 0.5 mL dose CIV H3N2 vaccine has been developed using a novel RNA Particle platform and confirmed to be both highly efficacious and safe in dogs as young as 8 weeks of age. An adjuvant is not needed in this vaccine formulation to induce a highly effective and robust immune response, as demonstrated by the significant reduction in lung consolidation and incidence of suppurative pneumonia in the vaccinated dogs compared to the placebo-vaccinated control dogs. In addition, a significant reduction in the duration and severity of clinical signs, as well as the duration and amount of virus shed post-challenge, was also demonstrated. The effectiveness of this new vaccine provides an opportunity to use this novel platform for other vaccine development programs to address unmet needs, improve the safety and efficacy profile of current vaccines, evaluate cross-protection to other strains, and quickly respond to future outbreaks of disease. 

## Data Availability

The datasets presented in this article are unavailable because the data are proprietary information of Merck Animal Health.
